# Association between the Prognostic Nutritional Index and Dietary Intake in Community-Dwelling Older Adults with Heart Failure: Findings from NHANES III

**DOI:** 10.3390/nu11112608

**Published:** 2019-10-31

**Authors:** Elisabeth L. P. Sattler, Yuta Ishikawa, Rupal Trivedi-Kapoor, Donglan Zhang, Arshed A. Quyyumi, Sandra B. Dunbar

**Affiliations:** 1 Department of Foods and Nutrition, College of Family and Consumer Sciences, University of Georgia, Athens, GA 30602, USA; 2 Department of Clinical and Administrative Pharmacy, College of Pharmacy, University of Georgia, Athens, GA 30602, USA; 3 Department of Health Policy and Management, College of Public Health, University of Georgia, Athens, GA 30602, USA; 4 Emory Clinical Cardiovascular Research Institute, Emory University School of Medicine, Atlanta, GA 30322, USA; 5 Nell Hodgson Woodruff School of Nursing, Emory University, Atlanta, GA 30322, USA

**Keywords:** heart failure, older adults, malnutrition, dietary intake, prognostic nutritional index

## Abstract

The objective of this study was to examine the association between nutritional status and dietary intake in community-dwelling older adults with heart failure (HF). A cross-sectional analysis of NHANES III data was conducted. The analytic sample was comprised of *n* = 445 individuals aged 50+ years with congestive HF (54.4% male, 22.9% non-Hispanic Black, 43.8% low-income). Nutritional status was measured using the Prognostic Nutritional Index (PNI). Participants were classified by PNI quintiles with lower PNI scores indicating lower nutritional status. Participants in quintile 5 showed significantly greater intakes of energy, protein, vegetables, magnesium, zinc, copper, potassium, red meat, saturated fat, and sodium. In multivariate analyses, increased intake of red meat (β = 0.253, *p* = 0.040) and vegetables (β = 0.255, *p* = 0.038) was associated with significantly better nutritional status. In the absence of comprehensive nutritional guidance for HF patients, it appears that small increases in energy, protein (red meat), and vegetable consumption are associated with improved nutritional status.

## 1. Introduction

Heart failure (HF) is a serious public health concern in the United States. More than six million Americans currently live with HF [1], and it is estimated that over eight million Americans will be diagnosed with HF by 2030 [2]. In older adults aged 65+ years, HF incidence is as high as 21 per 1000 [3], and prevalence rates increase from 6% in adults 60–79 years of age to 14% in adults 80+ years of age [1]. The progressive nature of HF deterioration causes functional limitations such as orthopnea, paroxysmal nocturnal dyspnea, fatigue and/or lethargy [4], all of which significantly reduce patient quality of life [5]. In addition to the human cost, total medical costs for HF were estimated to be $30.7 billion in 2012, about 80% of which were related to hospitalizations [2]. Older Americans are more often hospitalized for HF than for any other condition [6]; thus, with the aging of the population, HF-related medical costs are only expected to increase [2].

While pharmacological approaches are a cornerstone of HF management, non-pharmacological treatment strategies likely play an important role in the control of HF symptoms, reduction of hospital readmissions, and patient quality of life [7]. Dietary management of HF, a common non-pharmacological approach, has traditionally focused on sodium restriction alone, which is arguably the most heavily debated HF treatment strategy due to conflicting evidence leading to a lack of consensus on recommended sodium intake levels by professional societies and experts in the field [8,9]. While more research is needed to determine the role of sodium restriction in HF management, it may be important to acknowledge that malnutrition is highly prevalent in HF patients and is known to lead to poor HF prognosis and outcomes [10,11]. Older adults are predisposed to malnutrition due to age-related changes in cognition, physical and sensory function, medication interactions, social isolation, and low-socioeconomic status [12]. In HF, the situation is further complicated by the underlying pathophysiology of chronic inflammation and fluid overload, which lead to nausea, loss of appetite, and early satiety resulting from gastrointestinal edema and hepatic congestion [13]. Heart failure patients have been shown to have higher levels of total energy expenditure and hypercatabolic hormonal status, and are more likely to have negative energy and nitrogen balances compared to healthy controls, thus leading to energy-protein malnutrition [14]. In addition, they are less likely consuming adequate levels of energy to support increased energy requirements in a hypercatabolic state [14,15], and show inadequate dietary intakes of micronutrients, including vitamin B1, B2, B6, folate, vitamin C, D and E, calcium, magnesium, iron, selenium, coenzyme Q10, and creatine [16,17,18,19]. Consideration of malnutrition may be especially important in the context of sodium reduction recommendations endorsed by several clinical guidelines [9], as evidence suggests a relationship between low-sodium consumption and inadequate intakes of energy, macronutrients, and specific food groups [20,21].

Furthermore, HF pharmacotherapy has profound effects on nutritional status, but such effects are rarely considered within HF risk stratification and disease management approaches [8,22]. For example, angiotensin-converting-enzyme inhibitors, a key medication class for the treatment of HF in older adults through its effects on vasodilation, reduction of pre and afterload, and nephroprotective properties, may lead to taste distortions and reduced appetite [13,23]. Loop diuretics, often used for symptomatic management of HF exacerbations, contribute to micronutrient deficiencies, such as of B vitamins and minerals, including potassium, calcium, magnesium, and selenium through excessive urinary excretion [23]. Lastly, the nutritional status of older adults with HF is directly and indirectly affected by cardiac cachexia, defined as unintended, edema treatment-independent weight loss equal to at least 6% of body weight within 6–12 months, affecting a large proportion of malnourished HF patients [24]. Cardiac cachexia is a serious HF complication that is characterized by the loss of lean muscle, fat, and bone tissue, and is a risk factor for poor HF prognosis and mortality that is independent of HF severity, left ventricular ejection fraction, exercise capacity, and patient age [24]. The progression of cardiac cachexia is strongly associated with increasing levels of functional decline in older adults, and may, therefore, lead to an impaired ability to shop for and prepare meals [25]. 

The assessment of nutritional risk in older adults with HF is hampered by the lack of a universally accepted definition of and gold standard methodology for malnutrition; thus, varies by settings and assessment instruments [26,27]. Likewise, the prevalence of malnutrition among HF patients has a widely estimated range from 20 to 70% [28]. Due to the pathophysiological link between malnutrition and HF, recent studies have investigated the prognostic impact of novel nutritional status indices based on biochemical and clinical markers, including serum albumin, total lymphocyte count, and total cholesterol level [29,30]. Scores that represented poorer nutritional status were associated with significantly longer hospital stays, cardiovascular events, and mortality [29,30,31,32]. Although these clinical marker indices, including the Prognostic Nutritional Index (PNI) and the Controlling Nutritional Status (CONUT) scores, have been used in inpatient HF populations only, cardiac cachexia and protein-energy malnutrition are prevalent in community-dwelling chronic HF patients. Moreover, low albumin, triglyceride, and other clinical marker levels have been observed and linked to objectively assessed, validated nutritional risk in community-dwelling older adults with HF, such as the Mini Nutritional Assessment (MNA) [33].

Given the strong impact of poor nutritional status on HF prognosis and outcomes, and the potential for low-cost, dietary intervention for the early prevention of poor outcomes in community-dwelling older adults with HF, it is essential to better understand the relationship between nutritional status and dietary intake in this population. The objective of this study was, therefore, to examine the association between nutritional status and dietary intake in community-dwelling older adults with HF.

## 2. Materials and Methods

### 2.1. Study Design and Population

This study represents a secondary data analysis of survey data from the National Health and Nutrition Examination Survey (NHANES) III using a cross-sectional design. NHANES III was conducted by the National Center for Health Statistics between 1988 and 1994, and uses a multistate, stratified sampling design to include a nationally representative sample of non-institutionalized community-dwelling U.S. adults. The survey design and sampling methods have been described more in detail elsewhere [34].

### 2.2. Analytic Sample

The analytic sample included older adults 50+ years of age who self-reported “yes” to a query about HF (“Has a doctor ever told you that you had congestive heart failure?”). A total of *n* = 546 older adults with self-reported congestive HF completed NHANES interviews and Mobile Examination Center (MEC) visits. Individuals in the “other” racial/ethnic groups (*n* = 20) were excluded following the recommendation from the NHANES III Analytic and Reporting Guidelines [35]. Subjects with missing data on dietary intake (*n* = 23) and clinical parameters (serum albumin and lymphocyte levels) used for nutritional assessment (*n* = 54), and implausible reported energy intakes (<1st and >99th percentiles of energy intake per day, *n* = 31) were excluded from the analysis. The final sample included *n* = 445 older adults with self-reported congestive HF (54.4% male, 22.9% non-Hispanic Black, 59.8% currently married, 43.8% low-income). 

### 2.3. Variables

#### 2.3.1. Nutritional Status

The Prognostic Nutritional Index assesses nutritional status based on clinical marker values by using the following Equation (1) [29,30,31,32]:

(1) $$\mathrm{PNI}=10\;\times \;\mathrm{serum}\;\mathrm{albumin}\;\left(\frac{g}{\mathrm{dL}}\right)+0.005\;\times \;\mathrm{lymphocyte}\;\mathrm{count}\;\left({\mathrm{per}\;\mathrm{mm}}^{3}\right)$$

Relatively lower PNI scores indicate poorer nutritional status and were associated with an increased risk of rehospitalization and mortality in previous studies [29,30,31,32]. The specimen collection and processing procedures have been described elsewhere [36]. Levels of serum albumin may indicate protein reserve levels [37,38], and low total lymphocyte count may be associated with low immune function caused by undernutrition [39,40,41]. Participants in the analytic sample were classified by PNI quintiles, with participants in quintile 1 (lowest PNI scores) indicating a relatively higher risk of malnutrition whereas those in quintile 5 (highest PNI scores) indicating a relatively lower risk of malnutrition. 

#### 2.3.2. Dietary and Food Group Intake

Dietary intake was assessed via a single 24-hour dietary recall, using an automated, microcomputer-based dietary interview [42]. The food composition data for NHANES III was based on the U.S. Department of Agriculture (USDA) Survey Nutrient Database and the University of Minnesota’s Nutrition Coordinating Center. 

Food group intake was quantified using the Pyramid Servings Database (PSDB) for NHANES III, developed by the National Cancer Institute [43]. The PSDB facilitates serving size calculations based on the USDA Food Guide Pyramid’s food groups and includes 4542 different food item codes that correspond to all foods reported in the 24-hour dietary recalls. Major food groups in the USDA Food Guide Pyramid recommendations include dairy, fruit, vegetables, grains, and meat/other proteins [44]. Food group intake of fruit, vegetables, grains, and dairy was described using serving sizes, and intake of meat and other proteins was described using ounces or ounce lean meat equivalents. 

#### 2.3.3. Covariates

Socio-demographic and economic characteristics collected included age, gender, race/ethnicity (non-Hispanic White, non-Hispanic Black, and Mexican-American), education (<high school, high school, and >high school), working status, marital status, and poverty income ratio (PIR) (<1.300, 1.301–3.500, and ≥3.501). Health-related covariates included body mass index (BMI; kg/m^2^), systolic and diastolic blood pressure (mmHg), number of comorbidities (0–2, 3–4, 5+), number of hospitalizations in the past 12 months (never, 1, 2+), self-reported length of time since HF diagnosis (0–2, 3–5, 6–10, 11+ years), and clinical markers not included in the PNI score (levels of total cholesterol, triglycerides, HDL-cholesterol, and C-reactive protein (CRP)). In addition, we considered self-reported HF medication use, including diuretics (e.g., loop, thiazide, potassium-sparing diuretics), angiotensin-converting-enzyme inhibitors (ACEI), beta-blockers, digoxin, and nitrates (e.g., isosorbide mononitrate). Smoking status was defined by two questions (“Have you smoked at least 100 cigarettes during your entire life?” and “Do you smoke cigarettes now?”), and grouped into three categories (never, former, current smoker). Habitual drinking status was defined by two questions (“In your entire life, have you had at least 12 drinks of any kind of alcoholic beverage?” and “In the past 12 months did you have at least 12 drinks of any kind of alcoholic beverage?”), and if both answers were affirmative, a follow-up question asked about the number of drinks/ day on the days of alcohol consumption. According to the definition from the Substance Abuse and Mental Health Services Administration, if individuals drank 5+ drinks (men) or 4+ drinks (women) in one sitting, they were categorized as binge drinkers [45]. Physical activity (PA) levels were classified as active, insufficiently active, and inactive [46]. Individuals who reported 5+ episodes of moderate-intensity activity or 3+ episodes per week of vigorous-intensity activity in the past month were classified as active [47]. Those who reported some engagement of physical activity but did not meet the recommended levels in the previous month were classified as insufficiently active. 

### 2.4. Statistical Analysis

All statistical analyses were performed using SAS version 9.4 (SAS Institute Inc., Cary, NC, USA). The survey weights, which account for the complex survey sampling design of NHANES III, were not applied to the analyses because of the small size of the HF target population (e.g., larger variance). Participant characteristics were summarized using means and standard deviations (SD) for continuous variables and percentages for categorical variables. Univariate analysis of differences in sample characteristics by nutritional status was examined by *t*-tests or Mann-Whitney tests for continuous variables and by chi-squared tests for categorical variables. Linear regression analyses were conducted to generate beta coefficients and 95% confidence intervals (CIs) to quantify the association between nutritional status and sample characteristics while controlling for potential confounders. Data points influencing the estimated slope coefficient were excluded if Cook’s distance values were greater than 1.0. Multivariate analyses were used to assess the association between nutritional status and food group intake while adjusting for potentially confounding factors, as informed by univariate analysis using simple linear regressions. *p* values of < 0.05 were considered statistically significant.

## 3. Results

A total of *n* = 445 older adults with self-reported congestive HF were included in the analysis. In comparison to excluded individuals, included participants were more likely to be male (54.4 vs. 36.6%; *p* = 0.001), more educated (34.9 vs. 20.0% with high school diploma or higher; *p* = 0.016), currently working (15.7 vs. 4.0%; *p* = 0.001), married (59.8 vs. 46.5%; *p* = 0.015), and had higher incomes (56.2 vs. 40.5% with PIR > 1.300; *p* = 0.009).

Sample PNI score mean and ranges are shown in Table 1. Absolute PNI scores in the sample ranged from 32.2 to 73.0. Participants in quintile 1 had a mean PNI score of 43.8 ± 3.3 (mean ± SD), while those in quintile 5 had a mean score of 60.0 ± 3.5. Univariate analyses of sample descriptive characteristics by PNI quintiles are shown in Table 2. On average, the analytic sample was overweight (27.8 ± 5.7 kg/m^2^), had stage 2 hypertension (142 ± 22 mm Hg in systolic blood pressure), hyperlipidemia (224.0 ± 52.7 mg/dL total cholesterol; 189.2 ± 179.2 mg/dL triglycerides), and elevated levels of CRP (7.9 ± 14.0 mg/L). In comparison to participants in quintile 1, participants in quintile 5 were more likely male (64.0 vs. 49.4%; *p* = 0.049), currently smoking (23.6 vs. 12.4%; *p* = 0.041), and had been diagnosed with HF for a longer time period (35.3 vs. 16.1% with 6–10 years since 1st HF diagnosis; *p* = 0.012). In addition, those in quintile 5 were more likely to have higher levels of serum albumin (4.4 ± 0.4 vs. 3.6 ± 0.4 g/dL; *p* < 0.001), lymphocytes (3160 ± 919 vs. 1638 ± 535 per mm^3^; *p* < 0.001), total cholesterol (225.7 ± 37.6 vs. 216.7 ± 56.3 mg/dL; *p* = 0.042), triglycerides (208.5 ± 113.0 vs. 175.8 ± 229.3 mg/dL; *p* < 0.001), and lower levels of high-density lipoprotein cholesterol (HDL-C) (43.5 ± 12.8 vs. 52.0 ± 17.0 mg/dL; *p* < 0.001), CRP (6.0 ± 7.7 vs. 15.6 ± 26.2 mg/dL; *p* < 0.001), a smaller number of comorbidities (41.0 vs. 22.4% with 0–2 comorbidities; *p* = 0.027) and hospitalizations in the past 12 months (16.9 vs 24.4% with 2+ hospitalizations; *p* = 0.021), in comparison to those in the lowest quintile. 

Univariate analyses of sample dietary intake, including macro/micronutrients and food groups, by PNI scores are shown in Table 3. Participants in quintile 5 had higher intakes of energy (1629 ± 776 vs. 1356 ± 541 kcal; *p* = 0.037), protein (70.1 ± 41.0 vs. 58.6 ± 33.9 g; *p* = 0.039), saturated fat (20.9 ± 13.2 vs. 16.1 ± 9.5 g; *p* = 0.025), sodium (2611 ± 1380 vs. 2221 ± 1854 mg; *p* = 0.021), magnesium (268.1 ± 157.5 vs. 218.4 ± 117.3 mg; *p* = 0.034), zinc (10.9 ± 8.6 vs. 8.5 ± 6.7 mg; *p* = 0.008), copper (1.1 ± 0.7 vs. 0.9 ± 0.6 mg; *p* = 0.022), and potassium (2685.2 ± 1440.9 vs. 2130.4 ± 1029.9 mg; *p* = 0.010), than those in quintile 1. With regard to specific foods, individuals with higher PNI scores consumed 1.2 oz more red meat (2.4 ± 3.4 vs. 1.2 ± 1.5 oz; *p* = 0.028) and 1 more serving of vegetables (3.2 ± 2.8 vs. 2.2 ± 1.6 servings; *p* = 0.037), including 0.3 more servings of tomatoes (0.5 ± 0.6 vs. 0.2 ± 0.4 servings; *p* = 0.001) compared to individuals with lower PNI scores.

Results from linear regression analyses are presented in Table 4. In univariate analyses, higher PNI scores were associated with a higher red meat (*p* = 0.001) and vegetable intake (*p* = 0.004), smoking status (*p* = 0.033), and triglyceride levels (*p* = 0.011). Lower PNI score levels were associated with non-Hispanic Black race/ethnicity (*p* = 0.009), binge drinking (*p* = 0.033), number of comorbidities (*p* = 0.024), number of hospitalizations in the past 12 months (1 time, *p* = 0.029; 2+ times, *p* = 0.010), and levels of HDL-C (*p* = 0.001) and CRP (*p* < 0.001). In multivariate analyses, significant associations with PNI scores were found with red meat (*p* = 0.040) and vegetable intake (*p* = 0.038) after controlling for race/ethnicity, triglycerides, HDL-C, CRP, smoking status, binge drinking, and number of comorbidities and hospitalizations in the past 12 months. In both models, no collinearity among confounding factors was identified. Even after additionally controlling for gender and BMI in both models, the associations with PNI scores remained significant.

## 4. Discussion

To our knowledge, this is the first study to examine the relationship between nutritional status and dietary intake in community-dwelling older adults with congestive HF. Better nutritional status, as measured by albumin and lymphocyte levels, was associated with significantly higher intakes of energy, protein (specifically red meat), vegetables, and important macro- and micronutrients than in those with lower nutritional status in this population. 

In hospitalized patients with HF, poor nutritional status, as measured by clinical markers using the PNI, has been associated with higher levels of prognostic cardiometabolic markers, including brain naturetic peptide (BNP) and N-terminal pro BNP, indicating poor HF status and significantly longer hospital stays, cardiovascular events, and mortality [29,30,31,32,48]. In community-dwelling older adults, in whom regular blood collection is uncommon and inconvenient, subjective measures of nutritional status, including the MNA, have been validated and linked to adverse health outcomes, higher healthcare use, and mortality [49,50,51]. While the MNA measures nutritional risk due to a decline in food intake, unexpected weight loss, mobility, stress and psychological problems, and low BMI, MNA-assessed malnutrition has also been associated with significantly lower levels of albumin, total cholesterol, triglycerides, and higher levels of CRP. Given the links between HF pathophysiology, clinical markers of nutritional risk, and HF progression, application of these markers to community-dwelling, older HF patients holds strong potential for the identification and targeting of at-risk older adults with HF even before complications arise and patients are hospitalized.

Our sample of community-dwelling HF patients showed PNI scores ranging from 32.2 to 73.0, with participants in quintile 1 showing a mean PNI score of 43.8 ± 3.3 (mean ± SD), and those in quintile 5 showing a mean PNI score of 60.0 ± 3.5. While no standards have been established to evaluate the relationship of PNI scores with negative outcomes in community-dwelling HF patients, diverse thresholds have been suggested for hospitalized HF patients, including pre-determined scores (e.g., normal: PNI > 38; moderate risk of malnutrition: 35 < PNI < 38; and severe risk of malnutrition: PNI < 35) [30,31], receiver operating characteristic curves [29], and PNI tertiles [32]. Cheng and colleagues reported that acute HF patients at nutritional risk, as defined by PNI scores ≤44.8, had increased in-hospital, 90-day, and 4-year mortality rates [32].

On average, we found better nutritional status scores in our sample compared to average scores of hospitalized HF patients in previous studies. This is in line with the expectation of a more stable, community-dwelling HF population. In absolute terms, mean values for albumin (3.6 ± 0.4 g/dL; normal range: 3.5–5.5 g/dL) and lymphocyte count (1638 ± 535 in 1 µL; normal range: 1000–4800 in 1 µL) were at the lower end of the range of normal values. In relative terms, we found a proportion of patients in PNI quintile 1 to be at nutritional risk according to the threshold established by Cheng et al. in hospitalized HF patients. In addition, we found patients in PNI quintile 1 significantly more likely than patients in PNI quintile 5 to be hospitalized one or more times within the past year, which serves as a proxy for disease severity and is associated with increased mortality risk in HF patients [52]. Although only a small proportion of our sample fit the pre-determined criterion of nutritional risk, identifying nutritional characteristics of low nutritional status above thresholds associated with detrimental outcomes is likely important for the prevention of such events.

Applicability of dietary guidance for healthy individuals to HF patients is an ongoing debate; fueled in particular by conflicting evidence regarding the relationships between sodium intake and negative HF outcomes [9], discussion about adequate energy intake levels for HF [53], and the obesity paradox [54]. In the absence of comprehensive nutritional guidance for community-dwelling older adults with HF, more research is needed to determine how to best address nutritional deficiencies within complex HF pathophysiology and adverse effects of essential pharmacotherapy. In our sample of community-dwelling HF patients, total daily energy intake ranged from 1356 kcal (PNI quintile 1) to 1629 kcal (PNI quintile 5), and while adequacy of such levels based on predictive formulas was not assessed in this study, they are likely inadequate for a majority of participants in a sample with over 50% males, and as described in the previous literature [14,55]. Energy intake differed significantly by nutritional status, and this relationship was mainly driven by a difference in protein intake (58.6 ± 33.9 g vs. 70.1 ± 41.0 g) which is confirmed by results from previous studies [14,55]. Moreover, we found higher mean intakes of vegetables and red meat of ~1 serving and ~1 oz, respectively, to be significantly and independently associated with better nutritional status. Through these increases in intake, participants met food group intake recommendations for healthy adults at the time (3 servings of vegetables and 5 oz of protein, respectively) [44]. Therefore, our findings support the translation of food group recommendations for healthy adults to HF patients. There is growing evidence that protein intake, in particular, should be a focus of dietary HF management. Recent work from the BIOSTA-CHF trial in Europe showed that HF patients with higher intakes of protein (70+ g/day vs. ≤40 g/day) had significantly lower rates of mortality (31 vs. 18%; *p* < 0.001) [56]. These levels compare to our findings of the relationship between nutritional status and protein intake, and are supported by the significant relationship between dietary protein and serum albumin levels [57]. Furthermore, nutritional risk markers based on albumin levels have negative outcome predictive properties [57]. Interestingly, our findings suggest that heightened red meat intake (+1 oz/day), despite associated higher intake levels of saturated fat and sodium, as found in this study, may be protective of malnutrition. This finding suggests that in HF patients, particularly in those with comorbid hypertension and hyperlipidemia, long-term vs. short-term risks may need to be carefully evaluated. In this regard, it is likely that nutritional guidance for the prevention of cardiovascular events may not translate to HF patients at risk of malnutrition and cardiac cachexia. While past cardiovascular disease prevention research and Dietary Guidelines for Americans have recommended reduced intake of red meat due to concerns about cholesterol, red meat is a high quality source of essential amino acids and micronutrients, including thiamine, riboflavin, niacin, pyridoxine, vitamin B_12_, zinc, iron, selenium, copper, and magnesium, all of which are important for the nutritional well-being of older adults [58]. Grossniklaus and colleagues demonstrated that HF patients with higher consumption of protein, including red meat, had higher intakes of these micronutrients [59]. As a good source of magnesium, red meat may counter the loss of magnesium due to use of loop and thiazide diuretics, and may, therefore, counter symptoms of fatigue and ventricular ectopy, and improve HF prognosis [60,61]. Red meat consumption in older adults with HF may further increase muscle protein anabolism and reduce the progressive loss of muscle mass [62]. Therefore, adequate protein intake likely plays a crucial role in addressing the disease state-induced hypercatabolic state and in the prevention of cardiac cachexia. 

Increased consumption of vegetables has been associated with a lower risk of chronic diseases and mortality [63,64]. Vegetables are a well-known source of phytochemicals, antioxidants, vitamins, and minerals, which may play an important role in the prevention of malnutrition in HF patients [65]. The hyperadrenergic state, resulting from the compensation of decreased cardiac output experienced in HF [66], increases plasma free fatty acid (FFA) levels, which in turn, inhibit glycolysis and glucose uptake by the heart and skeletal muscle [67]. Increased plasma glucose promotes the formation of reactive oxygen species (ROS) which inhibit key enzymes for glycolysis [67]. In contrast, diets rich in phytochemicals and antioxidants may reduce levels of ROS as well as reduce levels of inflammatory markers, such as CRP [68], which we found elevated in those with lowest vegetable intake levels. Furthermore, there is evidence that increased lycopene intake from tomato products among HF patients is associated with longer cardiac event-free survival [69]. Our findings, therefore, support that vegetable consumption is important for older HF patients in the prevention of poor nutritional status.

Lastly, we found cigarette smoking to be positively associated with higher PNI values which is a controversial finding. Cigarette smoking is a well-established cardiovascular risk factor, and smoking status is a strong independent predictor of hospitalization and mortality in HF patients [70]. On the contrary, smoking cessation among HF patients significantly reduced the risks of hospitalization and mortality [70]. Despite these associations, clinical literature has documented a relationship between smoking status and elevated lymphocyte count [71] and higher energy intake [72]. Such evidence is in line with our findings and suggests smoking status as a potential confounding factor between dietary intake and PNI which we accounted for in our analyses. 

No study is without limitations. First, NHANES III is a cross-sectional study and causal inferences cannot be drawn. It is important to acknowledge that our findings reflect associations and may not be interpreted as causal relationships. In this context, it is possible that poor nutritional status, as shown by low PNI scores, may reflect more pronounced disease severity which may or may not be prevented by improved dietary intake. Furthermore, all self-reported information is subject to recall bias. Dietary information was collected by a single 24-hour dietary recall and may not represent habitual dietary intake of study participants. In addition, this study did not apply survey weights that adjust for biases arising from the complex sampling design used in NHANES III; therefore, the interpretation of the results should account for potential selection bias. Lastly, this study was limited in the adjustment for potentially confounding factors which may influence the association between dietary intake and clinically assessed nutritional status. For example, information on NYHA functional classification and other clinical characteristics of the HF disease state (e.g., reduced or preserved ejection fraction of left ventricular) were not available from the dataset. Moreover, we cannot exclude the possibility that any unmeasured factors may have led to improved survival in those with significantly better nutritional status, as we observed that individuals with better nutritional status had been diagnosed with HF for a longer amount of time. Nevertheless, this study provided valuable information for clinicians and patients on the relationship between nutritional status and dietary intake of energy and food groups. In addition, secondary data analysis of the NHANES III dataset had the advantage of using clinical and self-reported variables not available in other datasets (e.g., serum albumin and lymphocyte levels).

## 5. Conclusions

In the absence of comprehensive nutritional guidance for community-dwelling older adults with HF, it appears that small increases in energy, protein (red meat), and vegetable consumption are associated with an improved nutritional status which may prevent adverse events in this population. Controlled intervention studies are needed to determine if nutritional risk in HF patients can be minimized by the intake of specific dietary patterns.

## Figures and Tables

**Table 1:** Description of Prognostic Nutritional Index Scores in the Analytic Sample.

PNI ^1^ Quintile	PNI Score	
	Mean ± SD	Range
Total (*n* = 445)	51.5 ± 5.9	32.2–73.0
Quintile 1 (*n* = 89)	43.8 ± 3.3	32.2–47.1
Quintile 2 (*n* = 89)	48.7 ± 0.8	47.2–49.9
Quintile 3 (*n* = 89)	51.1 ± 0.7	49.9–52.5
Quintile 4 (*n* = 89)	54.1 ± 1.0	52.5–56.1
Quintile 5 (*n* = 89)	60.0 ± 3.5	56.1–73.0

^1^ PNI indicates Prognostic Nutritional Index.

**Table 2:** Characteristics of the Analytic Sample by Prognostic Nutritional Index Quintiles.

Characteristics	Total (*n* = 445)	Prognostic Nutritional Index					*p*-Value
		≤47.1 (*n* = 89)	47.1 to 49.9 (*n* = 89)	49.9 to 52.5 (*n* = 89)	52.5 to 56.1 (*n* = 89)	≥56.1 (*n* = 89)	
Age, years	70.6 ± 9.6	70.9 ± 9.2	71.6 ± 9.4	70.7 ± 10.3	71.1 ± 8.8	68.6 ± 10.2	0.123 ^a^
Male, %	54.4	49.4	48.3	53.9	56.2	64.0	0.049
Race/Ethnicity, %							
Non-Hispanic White	47.6	41.6	47.2	43.8	56.2	49.4	0.137
Non-Hispanic Black	22.9	28.1	25.8	27	18	15.7	
Mexican-American	29.4	30.3	27.0	29.2	25.8	34.8	
Education, %							
Less than High School	65.2	76.1	61.4	60.9	63.6	63.6	0.139
High School Graduate	19.4	11.4	20.5	25.3	18.2	21.6	
More than High School	15.5	12.5	18.2	13.8	18.2	14.8	
Currently working, %	15.7	14.6	16.9	19.1	10.1	18.0	0.543
Currently married, %	59.8	57.3	59.1	60.2	60.7	61.8	0.541
Poverty Income Ratio, %							
≤1.300	43.8	43.0	40.5	47.3	43.2	45.5	0.159
1.301–3.500	42.3	46.8	46.4	39.2	43.2	35.1	
≥3.501	13.9	10.1	13.1	13.5	13.5	19.5	
Body Mass Index, kg/m^2^	27.8 ± 5.7	27.8 ± 6.8	27.6 ± 6.2	27.6 ± 5.5	28.2 ± 5.5	27.9 ± 4.1	0.186 ^a^
Systolic Blood Pressure, mm Hg	142 ± 22	140 ± 21	139.9 ± 6	142.7 ± 22	143.6 ± 21	142 ± 20	0.583 ^b^
Diastolic Blood Pressure, mm Hg	73 ± 12	74 ± 11	72.2 ± 12	74.3 ± 13	72.5 ± 11	74 ± 12	0.878 ^b^
Clinical Biomarkers							
Total Cholesterol, mg/dL	224.0 ± 52.7	216.7 ± 56.3	223.1 ± 55.0	230.0 ± 274.5	224.7 ± 41.7	225.7 ± 37.6	0.042 ^a^
Triglycerides, mg/dL	189.2 ± 179.2	175.8 ± 229.3	175.9 ± 117.2	203.3 ± 274.5	182.6 ± 84.3	208.5 ± 113.0	<0.001 ^a^
HDL-Cholesterol, mg/dL	48.3 ± 15.8	52.0 ± 17.0	49.5 ± 15.9	51.0 ± 16.9	45.3 ± 14.8	43.5 ± 12.8	<0.001 ^a^
C-reactive Protein, mg/L	7.9 ± 14.0	15.6 ± 26.2	7.0 ± 9.5	5.8 ± 6.1	5.3 ± 6.5	6.0 ± 7.7	<0.001 ^a^
Albumin, g/dL	4.0 ± 0.4	3.6 ± 0.4	3.9 ± 0.2	4.0 ± 0.2	4.2 ± 0.3	4.4 ± 0.4	<0.001 ^a^
Lymphocyte Count /mm^3^	2244 ± 807	1638 ± 535	1898 ± 499	2127 ± 494	2398 ± 530	3160 ± 919	<0.001 ^a^
Smoking, %							
Never	38.2	43.8	40.5	40.5	38.2	28.1	0.041
Former	46.1	43.8	47.2	44.9	44.9	48.3	
Current	15.7	12.4	12.4	14.6	15.7	23.6	
Binge Drinking, %	5.2	12.8	1.2	3.5	3.5	4.7	0.102
Physical Activity, %							
Inactive	44.7	44.9	49.4	49.4	50.6	29.2	0.094
Insufficiently Active	31.0	33.7	25.8	28.1	24.7	42.7	
Active	24.3	21.4	24.7	24.7	24.7	28.1	
Number of Comorbidities, %							
0–2	30.2	22.4	29.2	29.2	28.7	41.0	0.027
3–4	39.0	49.4	37.1	43.7	31.0	33.7	
5+	30.9	28.2	33.7	26.4	40.2	25.3	
Years since 1st HF Diagnosis, %							
0–2 years	24.8	25.3	20.9	32.9	21.6	23.5	0.012
3–5 years	19.5	27.6	18.6	17.7	20.5	12.9	
6–10 years	24.8	16.1	30.2	21.2	21.6	35.3	
11+ years	30.9	31.0	30.2	28.2	36.4	28.2	
Number of Hospitalizations in the Past 12 Months, %							
Never	62.9	53.5	61.8	62.5	63.2	73.0	0.021
1	19.4	22.1	22.5	18.2	24.1	10.1	
2+	17.8	24.4	15.7	19.3	12.6	16.9	
Medication Use							
Diuretics, %	46.4	49.4	43.9	54.6	38.6	46.1	0.680
ACEI, %	24.4	19.0	26.8	29.9	27.7	18.4	0.928
Beta-Blockers, %	14.9	13.9	11.0	16.9	16.9	15.8	0.744
Digitoxin, %	28.2	25.3	29.3	29.3	30.1	26.3	0.887
Isosorbide, %	12.9	16.5	11.0	15.6	9.6	11.8	0.411

HDL-Cholesterol: high-lipoprotein density cholesterol; HF: heart failure; ACEI: angiotensin-converting-enzyme inhibitors. Continuous variables are shown as means ± standard deviation. *p*-values show the difference between participants with the lowest and highest prognostic nutritional index scores, resulting from *t*-tests or Mann-Whitney U tests for continuous, and chi-square tests for categorical variables. ^a^ Results from *t*-test. ^b^ Results from Mann-Whitney U test.

**Table 3:** Nutritional Intake in the Analytic Sample by Prognostic Nutritional Index Quintiles.

Nutritional Intake	Total (*n* = 445)	Prognostic Nutritional Index					*p*-Value
		≤47.1 (*n* = 89)	47.1 to 49.9 (*n* = 89)	49.9 to 52.5 (*n* = 89)	52.5 to 56.1 (*n* = 89)	≥56.1 (*n* = 89)	
Total Calories, kcal	1537 ± 693	1356 ± 541	1465 ± 626	1620 ± 715	1613 ± 751	1629 ± 776	0.037
**Macronutrients**							
Protein, g	63.7 ± 32.2	58.6 ± 33.9	57.4 ± 24.0	67.4 ± 29.5	65.2 ± 28.8	70.1 ± 41.0	0.039
Total Energy from Protein, % kcal from Protein	17.0 ± 4.9	17.4 ± 5.6	16.3 ± 4.7	17.2 ± 5.1	16.8 ± 4.7	17.3 ± 4.3	0.762
Total Fat, g	56.3 ± 32.5	48.4 ± 28.0	53.9 ± 27.5	57.4 ± 33.7	59.9 ± 32.2	62.0 ± 38.8	0.050
Total Energy from Total Fat, % kcal from Total Fat	32.2 ± 9.3	31.2 ± 10.1	32.7 ± 8.0	31.3 ± 10.2	33.2 ± 7.9	32.7 ± 10.0	0.270
Saturated Fatty Acids, g	18.7 ± 11.1	16.1 ± 9.5	17.4 ± 9.7	19.2 ± 11.8	19.7 ± 10.5	20.9 ± 13.2	0.025
Monounsaturated Fatty Acids, g	21.4 ± 13.5	18.6 ± 12.5	20.0 ± 10.6	21.9 ± 14.1	23.0 ± 13.8	23.7 ± 15.7	0.059
Polyunsaturated Fatty Acids, g	11.6 ± 8.3	9.7 ± 6.3	12.2 ± 7.8	11.5 ± 8.1	12.4 ± 8.6	12.2 ± 10.3	0.218
Carbohydrates, g	196.8 ± 95.5	176.8 ± 69.9	188.0 ± 87.2	214.7 ± 112.7	204.5 ± 108.1	200.1 ± 91.1	0.182
Total Energy from Carbohydrates, % kcal from Carbohydrates	51.7 ± 11.6	52.8 ± 12.4	51.4 ± 11.1	53.1 ± 12.1	50.7 ± 10.5	50.8 ± 11.7	0.153
Cholesterol, mg	256.6 ± 194.2	229.6 ± 174.4	222.6 ± 160.4	263.2 ± 182.6	273.1 ± 193.6	294.5 ± 244.2	0.061
Dietary Fiber, g	15.1 ± 10.8	12.7 ± 8.0	14.5 ± 10.0	16.2 ± 11.5	15.5 ± 10.8	16.5 ± 12.9	0.072
**Micronutrients**							
Vitamin A, IU	6966 ± 9911	6561 ± 9677	7656 ± 9717	6712 ± 10452	6347 ± 9086	7552 ± 10690	0.515
Thiamin, mg	1.43 ± 0.87	1.29 ± 0.77	1.39 ± 0.80	1.47 ± 0.91	1.41 ± 0.75	1.59 ± 1.06	0.133
Riboflavin, mg	1.71 ± 0.98	1.56 ± 0.95	1.70 ± 0.97	1.72 ± 0.97	1.72 ± 0.92	1.84 ± 1.06	0.067
Niacin, mg	18.2 ± 11.3	17.2 ± 14.0	17.0 ± 9.4	19.3 ± 10.2	17.6 ± 9.3	20.0 ± 12.7	0.080
Vitamin B12, µg	4.2 ± 5.1	4.1 ± 7.4	4.3 ± 6.1	4.0 ± 3.6	4.2 ± 3.5	4.3 ± 4.0	0.124
Folate, µg	264.7 ± 212.0	227.9 ± 204.1	263.3 ± 185.8	280.9 ± 228.8	265.6 ± 206.3	285.8 ± 231.5	0.065
Vitamin C, mg	100.8 ± 99.6	91.6 ± 81.5	111.3 ± 124.5	106.5 ± 104.2	91.7 ± 77.3	103.1 ± 103.8	0.588
Vitamin D, µg	4.3 ± 3.8	4.0 ± 3.0	4.5 ± 3.9	4.5 ± 4.3	4.4 ± 3.7	4.4 ± 4.0	0.963
Vitamin E, mg α-tocopherol equiv.	7.6 ± 10.0	7.0 ± 12.0	7.5 ± 10.6	7.6 ± 9.1	8.2 ± 8.7	7.9 ± 9.4	0.139
Calcium, mg	653.4 ± 436.6	607.2 ± 350.6	647.4 ± 443.1	685.0 ± 507.0	661.7 ± 435.7	665.9 ± 438.4	0.736
Magnesium, mg	245.5 ± 131.8	218.4 ± 117.3	231.9 ± 118.0	262.2 ± 134.3	246.9 ± 124.2	268.1 ± 157.5	0.034
Iron, mg	13.4 ± 10.1	11.4 ± 8.4	13.6 ± 11.9	13.9 ± 9.7	13.8 ± 9.1	14.1 ± 11.1	0.095
Zinc, mg	9.8 ± 7.0	8.5 ± 6.7	9.1 ± 6.2	10.6 ± 7.3	10.2 ± 5.7	10.9 ± 8.6	0.008
Copper, mg	1.0 ± 0.6	0.9 ± 0.6	1.0 ± 0.5	1.1 ± 0.5	1.1 ± 0.5	1.1 ± 0.7	0.022
Potassium, mg	2418.9 ± 1208.8	2130.4 ± 1029.9	2338.9 ± 1131.6	2575.9 ± 1266.6	2364.2 ± 1077.1	2685.2 ± 1440.9	0.010
Phosphorus, mg	1025.2 ± 517.7	957.4 ± 505.0	945.7 ± 473.9	1059.4 ± 511.4	1051.0 ± 490.5	1112.2 ± 591.6	0.076
Sodium, mg	2485 ± 1457	2221 ± 1854	2361 ± 1294	2595 ± 1305	2634 ± 1363	2611 ± 1380	0.021
**Food Groups (No. of Servings)**							
Fruit	1.7 ± 2.3	1.7 ± 2.1	2.0 ± 3.1	1.7 ± 2.2	1.6 ± 1.6	1.6 ± 2.1	0.513
Vegetables	2.8 ± 2.4	2.2 ± 1.6	2.7 ± 2.5	3.1 ± 2.6	2.7 ± 2.1	3.2 ± 2.8	0.037
Deep Yellow Vegetables	0.2 ± 0.5	0.2 ± 0.4	0.2 ± 0.3	0.2 ± 0.5	0.2 ± 0.5	0.2 ± 0.6	0.614
Dark Green Leafy Vegetables	0.2 ± 0.8	0.2 ± 0.7	0.3 ± 1.2	0.3 ± 0.8	0.2 ± 0.6	0.1 ± 0.4	0.772
Bean and Peas	0.3 ± 0.9	0.1 ± 0.5	0.2 ± 0.5	0.4 ± 1.1	0.4 ± 1.0	0.4 ± 1.0	0.087
Tomatoes	0.4 ± 0.7	0.2 ± 0.4	0.4 ± 1.0	0.5 ± 0.7	0.4 ± 0.7	0.5 ± 0.6	0.001
Other Vegetables	0.9 ± 1.1	0.8 ± 0.9	0.9 ± 1.4	0.9 ± 1.2	0.8 ± 0.9	1.0 ± 1.1	0.067
Grains	5.3 ± 3.0	5.0 ± 2.5	5.1 ± 2.8	5.4 ± 2.9	5.4 ± 3.2	5.4 ± 3.5	0.970
Whole Grains	1.0 ± 1.6	1.1 ± 1.6	1.0 ± 1.3	1.2 ± 1.9	1.1 ± 1.7	0.8 ± 1.4	0.281
Meat and Other Proteins, oz	4.2 ± 3.1	3.8 ± 3.1	3.7 ± 2.4	4.5 ± 3.1	4.3 ± 2.8	4.8 ± 3.9	0.071
Red Meat, oz	1.6 ± 2.3	1.2 ± 1.5	1.3 ± 1.7	1.6 ± 2.4	1.5 ± 2.1	2.4 ± 3.4	0.028
Poultry, oz	1.0 ± 2.0	1.3 ± 2.5	0.8 ± 1.6	1.3 ± 2.1	1.1 ± 2.0	0.8 ± 1.7	0.080
Fish and Other Seafood, oz	0.4 ± 1.3	0.4 ± 1.1	0.5 ± 1.6	0.6 ± 1.3	0.4 ± 1.3	0.2 ± 0.8	0.258
Eggs, oz lean meat equivalents	0.6 ± 0.8	0.5 ± 0.7	0.5 ± 0.7	0.6 ± 0.8	0.6 ± 0.9	0.6 ± 0.9	0.938
Luncheon Meats, oz	0.5 ± 1.0	0.3 ± 0.7	0.5 ± 0.9	0.4 ± 0.9	0.6 ± 1.1	0.6 ± 1.2	0.063
Nuts and Seeds, oz lean meat equivalents	0.1 ± 0.4	0.1 ± 0.3	0.1 ± 0.2	0.1 ± 0.2	0.1 ± 0.3	0.1 ± 0.6	0.635
Soy Product, oz lean meat equivalents	0.0 ± 0.2	0.0 ± 0.0	0.1 ± 0.5	0.0 ± 0.1	0.0 ± 0.0	0.0 ± 0.0	0.557
Dairy	1.1 ± 1.2	1.2 ± 1.1	1.2 ± 1.3	1.1 ± 1.2	1.1 ± 1.1	1.1 ± 1.3	0.368

Continuous variables are shown as mean ± standard deviation. *p*-values, obtained from the Mann-Whitney U test, show the difference between participants with the lowest and highest prognostic nutritional index scores.

**Table 4:** Correlation of the Prognostic Nutritional Index by Univariate and Multivariate Linear Regression Analyses.

Parameters	Univariate			Multivariate					
				Model 1^a^ (R^2^ = 0.140)			Model 2 ^b^ (R^2^ = 0.141)		
	β	CI	*p*-Value	β	CI	*p*-Value	β	CI	*p*-Value
Red Meat	0.404	(0.174, 0.635)	0.001	0.253	(0.012, 0.492)	0.040			
Vegetables	0.335	(0.107, 0.564)	0.004				0.255	(0.014, 0.496)	0.038
Race/Ethnicity									
Non-Hispanic White	Ref.			Ref.			Ref.		
Non-Hispanic Black	−1.833	(−3.210, −0.455)	0.009	−1.278	(−2.738, 0.182)	0.086	−1.013	(−2.505, 0.479)	0.183
Mexican-American	0.065	(−1.206, 1.335)	0.920	−0.040	(−1.368, 1.289)	0.953	0.200	(−1.157, 1.557)	0.773
Clinical Biomarkers									
Triglycerides	0.006	(0.001, 0.010)	0.011	0.002	(−0.003, 0.006)	0.499	0.002	(−0.003, 0.006)	0.430
HDL-Cholesterol	−0.057	(−0.091, −0.022)	0.001	−0.050	(−0.085, −0.010)	0.013	−0.047	(−0.084, −0.009)	0.015
Serum C-reactive Protein	−0.143	(−0.192, −0.094)	<0.001	−0.091	(−0.129, −0.053)	<0.001	−0.097	(−0.134, −0.059)	<0.001
Smoking									
Never	Ref.			Ref.			Ref.		
Former	0.944	(−0.245, 2.134)	0.120	0.546	(−0.662, 1.753)	0.375	0.519	(−0.689, 1.727)	0.399
Current	1.770	(0.141, 3.398)	0.033	1.602	(−0.087, 3.291)	0.063	2.041	(0.375, 3.708)	0.017
Binge Drinking	−2.750	(−5.271, −0.230)	0.033	−1.768	(−4.360, 0.823)	0.181	−1.791	(−4.383, 0.801)	0.175
Number of Comorbidities									
0–2	Ref.			Ref.			Ref.		
3–4	−1.517	(−2.831, −0.203)	0.024	−1.445	(−2.781, −0.108)	0.034	−1.338	(−2.681, 0.005)	0.051
5+	−1.143	(−2.530, 0.244)	0.106	−0.894	(−2.369, 0.581)	0.234	−0.676	(−2.173, 0.820)	0.375
Number of Hospitalizations in the Past 12 Months									
Never	Ref.			Ref.			Ref.		
1	−1.579	(−0.162, −3.00)	0.029	−0.715	(−2.143, 0.712)	0.325	−0.715	(−2.143, 0.712)	0.325
2+	−1.940	(−0.475, −3.40)	0.010	−1.089	(−2.532, 0.355)	0.139	−1.049	(−2.493, 0.396)	0.154

HDL-cholesterol indicates high-density lipoprotein cholesterol. ^a^ Multivariate linear regression analysis of red meat intake adjusted for race/ethnicity, triglycerides, HDL-cholesterol, C-reactive protein, smoking status, binge drinking, number of comorbidities, and number of hospitalizations in the past 12 months. ^b^ Multivariate linear regression analysis of vegetable intake adjusted for race/ethnicity, triglycerides, HDL-cholesterol, C-reactive protein, smoking status, binge drinking, number of comorbidities, and number of hospitalizations in the past 12 months.
